# Cloning, expression, and immunocharacterization of surface protein containing an altered thrombospondin repeat domain (SPATR) from *Plasmodium knowlesi*

**DOI:** 10.1186/1475-2875-12-182

**Published:** 2013-06-04

**Authors:** Vanitha Palaeya, Yee Ling Lau, Rohela Mahmud, Yeng Chen, Mun Yik Fong

**Affiliations:** 1 Department of Parasitology, TIDREC, Faculty of Medicine, University of Malaya, 50603 Kuala Lumpur, Malaysia

**Keywords:** Malaria, Detection, *Plasmodium knowlesi*, ELISA, Surface protein containing an altered thrombospondin repeat domain (SPATR)

## Abstract

**Background:**

*Plasmodium knowlesi* is the fifth species identified to cause malaria in humans and is often misdiagnosed as *Plasmodium malariae* due to morphological similarities. The development of an inexpensive, serological detection method utilizing antibodies specific to *P. knowlesi* would be a valuable tool for diagnosis. However, the identification of specific antigens for these parasites remains a major challenge for generating such assays. In this study, surface protein containing an altered thrombospondin repeat domain (SPATR) was selected as a potentially specific antigen from *P. knowlesi*. Its multistage expression by sporozoites, asexual erythrocytic forms and gametocytes, along with its possible role in liver cell invasion, suggests that SPATR could be used as a biomarker for diagnosis of *P. knowlesi*.

**Methods:**

The *spatr* gene from *P. knowlesi* was codon optimized and cloned (*pkhspatr*). Recombinant *pk*HSPATR protein was expressed, purified, and evaluated for its sensitivity and specificity in immunoblot and ELISA-based assays for detecting *P. knowlesi* infection.

**Results:**

The recombinant *pk*HSPATR protein allows sensitive detection of human *P. knowlesi* infection in serum samples by immunoblot and ELISA.

**Conclusions:**

With further research, recombinant *pk*HSPATR protein could be exploited as a marker for detection of *P. knowlesi* infection in humans. Therefore, this finding should contribute to the development of immunodiagnostic assays for the species-specific detection of malaria.

## Background

Malaria remains a major health threat in many parts of the world. In 2012, the World Malaria Report by the World Health Organization (WHO) reported an estimated 219 million cases of malaria and 660,000 deaths for the year 2010. Emergence of *Plasmodium knowlesi* as the fifth human malaria species has raised the concern of scientists on the prospect for malaria elimination [1]. The *P. knowlesi* parasites have a 24-hour life cycle, which results in rapid amplification of parasitaemia. *P. knowlesi* infection is fast spreading and potentially dangerous, which makes accurate diagnosis vital for effective treatment of this parasitic disease. However, *P. knowlesi* parasites are often misdiagnosed as *Plasmodium malariae* due to their morphological similarities [2]. Use of polymerase chain reaction (PCR) to detect *P. knowlesi* infection is also not very accurate as the species-specific primers have been reported to crossreact with *Plasmodium vivax*[3] due to the close phylogenetic relationship of these two species. Thus, the development of an inexpensive, serological detection method utilizing antibodies specific to *P. knowlesi* would be a valuable tool for diagnosis of knowlesi malaria. Increased understanding on the parasite biology and putative biological function of malarial genes, due to recent advances in bio-informatics has been very helpful in evaluating new antigens as potential diagnostic or vaccine candidates.

The phylum Apicomplexa comprises a large group of protists, including *Babesia*, *Plasmodium*, *Cryptosporidium* and *Toxoplasma*, which are characterized by specialized secretory organelles found at their apical prominence. These apical structures, consisting of micronemes, rhoptries, and dense granules [4], contain many proteins that are involved in parasite motility and host cell invasion. Many invasive stages of *Plasmodium* have been found to contain one or more thrombospondin type-1 repeat (TSR) domain [5], suggesting the possible role of this domain in sporozoite motility and hepatocyte invasion. An altered version of the thrombospondin repeat domain was first found in *Plasmodium yoelii*[6,7] and named the surface protein of altered thrombospondin repeat (SPATR). Orthologue of this protein has also been identified in *P. knowlesi* and named the *pk*SPATR.

SPATR protein is expressed during many stages of the *P. knowlesi* life cycle, including the sporozoite, merozoite and gametocyte stages [6]. Importantly, it is expressed on the cell surface during the sporozoite stage, where it is involved in liver cell invasion. In fact, it was previously demonstrated that recombinant SPATR from *Plasmodium falciparum* and its orthologue in *P. knowlesi* were able to bind to HepG2 cells with high specificity, and antibodies generated against SPATR could inhibit *P. falciparum* sporozoite invasion into liver cells [6,7]. The *P. falciparum* SPATR protein was recognized by sera from naturally infected Africans but not control sera from two non-immune donors, indicating that this protein was recognized by the host immune system [6].

In this study, the *P. knowlesi spatr* gene sequence was codon optimized and cloned (*pkhspatr*). Moreover, recombinant *pk*HSPATR protein was expressed, purified, and evaluated for its sensitivity and specificity in immunoblot- and ELISA-based assays for detecting *P. knowlesi* infection in human serum samples.

## Methods

### Polymerase chain reaction (PCR) and cloning

The *P. knowlesi spatr* gene sequence was identified in a BLAST search of the *P. knowlesi* database using the published sequence of *P. falciparum spatr* [GenBank: AY952327] as a query sequence. The *spatr* sequence was codon optimized by Epoch Life Science Inc (USA) in order to enhance expression in *Escherichia coli*. The synthetic gene (806 base pairs, full length) was designated as *pkhspatr* and was cloned into SmaI digested pBluescript II SK. Forward (5′-GAATTCAAAA GAATGAAGAAG-3′) and reverse (5′-GAATTCGCCGTTTTGGTTAGTAG-3′) primers were used to PCR amplify *pkhspatr*, which was then cloned into pGEM-T vector (Invitrogen, USA). *EcoRI* restriction sites were introduced into both the forward and reverse primers in order to facilitate cloning. The *pkhspatr* gene was then digested with *Eco*RI restriction enzyme and cloned into the T7 promoter-based pRSET B vector, which allows expression of dually polyhistidine (His)- and Xpress epitope-tagged (both at the N-terminus) recombinant proteins in *E. coli* (Invitrogen, USA). The nucleotide sequence was analysed by NHK Bioscience Solutions Sdn Bhd (Malaysia).

### Expression of recombinant *pk*HSPATR

For protein expression in *E. coli*, the *pkhspatr*-pRSET B recombinant plasmid was transformed into Rosetta DE3 competent cells (Novagen, USA). Transformants were plated onto Luria-Bertani (LB) agar plates, which were supplemented with ampicillin (100 μg/ml) and chloramphenicol (32 μg/ml). A single, positive transformed colony was inoculated for expression of recombinant SPATR protein. After the culture reached an optical density at 600 nm of 0.6, expression of the *pkhspatr* gene was induced with 1 mM isopropyl-1-thio-D-galactopyranoside (IPTG) for four hours. Cells were then pelleted at 5,000 rpm for 10 min, the supernatant was discarded, and the wet weight of the pellet was determined. The pellet was then resuspended in 5 ml of Bug Buster reagent (Novagen, USA) per gram of wet pellet, and 1 μl of Benzonase (50 mM Tris-HCl, pH 8.0, 20 mM NaCl, and 2 mM MgCl_2_ in 50% glycerol) was added per ml of Bug Buster reagent used. Insoluble cell debris were separated through centrifugation at 16,000 x g for 20 min at 4°C, and the pellet was further lysed using 200 μg/ml lysozyme in the same volume of Bug Buster reagent that had been used during pellet resuspension. The cell suspension was then centrifuged at 16,000 × g for 15 min at 4°C, and the resulting pellet was repeatedly washed with 1:10 diluted Bug Buster reagent. After three washes, the final inclusion body-containing pellet was resuspended in phosphate buffered saline (PBS), and a small fraction was saved for sodium dodecyl sulfate-polyacrylamide gel electrophoresis (SDS-PAGE) and western blot analysis.

### Purification of recombinant *pk*HSPATR

Recombinant *pk*HSPATR protein was resuspended in 8 ml of 6 M urea and added to a ProBond column (Invitrogen, USA). The column was placed on a MACSmix™ Tube Rotator (Miltenyi Biotec, Germany), and the His-tagged protein was allowed to bind to the Ni-NTA agarose for four hours at 4°C. Once the resin had settled, the supernatant was gently aspirated, and the column was washed with 8 ml of 6 M urea. This step was repeated with 4 M and 2 M urea. Finally the protein was eluted with 1 ml of 1 M urea and 250 mM imidazole. The purity of recombinant *pk*HSPATR was determined using a 12% SDS-PAGE gel. The purified protein was then dialysed against PBS overnight at 4°C with two buffer changes during the dialysis period. The final purified *pk*HSPATR protein was quantified using the Bradford Assay Kit (Bio-Rad, USA).

### Gel electrophoresis and western blot

Proteins were separated according to their molecular weight using a 12% SDS-PAGE gel and transferred by electroblotting onto a polyvinylidene difluoride (PVDF) membrane (Bio-Rad, USA). The membranes were blocked overnight at 4°C using blocking solution (5% [w/v] skim milk in 1X Tris-buffered saline [TBS]). They were then washed twice using 1X TBS with 0.01% Tween 20 (TBST), followed by two washes with 1X TBS. After incubation with primary antibody (anti-Xpress antibody, 1:5,000 dilution, Invitrogen, USA; or human serum, 1:200 dilution) in TBS with 2.5% (w/v) skim milk at room temperature for two hours, the membrane was washed twice with both TBST and TBS. Bound antibodies were incubated with secondary antibody, biotin-labelled goat anti-mouse IgG (for anti-Xpress antibody) or biotin- labelled goat anti-human IgG (for human serum) (1:2,500 dilution) (Kirkegaard and Perry Laboratories, USA). This step was followed by incubation for an hour at room temperature with streptavidin–alkaline phosphatase (AP) (1:2,500 dilution) (Invitrogen, USA) and the standard washing step. Protein band was visualized using 5-bromo-4-chloro-3-indolyl phosphate and nitroblue tetrazolium (Sigma Chemical Co, USA).

### ELISA

A 96-well ELISA plate was coated with 10 μg of *pk*HSPATR recombinant protein and incubated overnight at 4°C. The next day, the plate was washed three times using PBS with 0.01% Tween 20 (PBST) and blocked with 1% BSA (Bovine Serum Albumin) in PBS for two hours at 37°C. Following three washes with PBST, the plate was incubated with patient serum (1:80 dilution) for one hour at 37°C, washed five times with PBST, and incubated with HRP-conjugated goat anti-human lgG (1:2,500 dilution) at 37°C for one hour. The plate was then washed vigorously with PBST (five times), and 3,3′,5,5′-tetramethylbenzidine (TMB) was used for the development reaction (20 min incubation in the dark). Optical density was read at 450 nm using an Infinite 200 PRO plate reader (TECAN, Switzerland).

### Sensitivity and specificity evaluation of recombinant *pk*HSPATR

Purified recombinant protein was further evaluated through western blot and ELISA using human serum samples obtained from Diagnostic Laboratory (Para: SEAD), Department of Parasitology, University of Malaya. The serum samples were categorized into four groups according to previous diagnosis using PCR and microscopy: (i) *P. knowlesi*; (ii) non-*knowlesi Plasmodium*; (iii) healthy donors (88 samples); and (iv) non-malaria parasitic infections, including *Toxoplasma gondii* (14 samples), amoebiasis (three samples), cysticercosis (two samples), and filariasis (two samples). In addition, 11 malaria negative samples, which were initially suspected as malaria infection, were included within the other infections group. The cut-off value for ELISA was calculated based on the healthy donor samples (mean + 2SD).

## Results

### PCR and cloning

The codon optimized *pkhspatr* gene was successfully amplified (806 bp) and cloned into the pRSET B vector, which allows bacterial expression of dually His- and Xpress epitope-tagged recombinant proteins. The *pkhspatr*-pRSET B plasmid was then isolated and sequenced. Sequence analysis indicated a complete match with the published amino acid sequence of Mahajan [6]. The plasmid was then transformed into Rosetta DE3 competent *E. coli* cells.

### Expression of recombinant *pk*HSPATR

**Figure 1:**
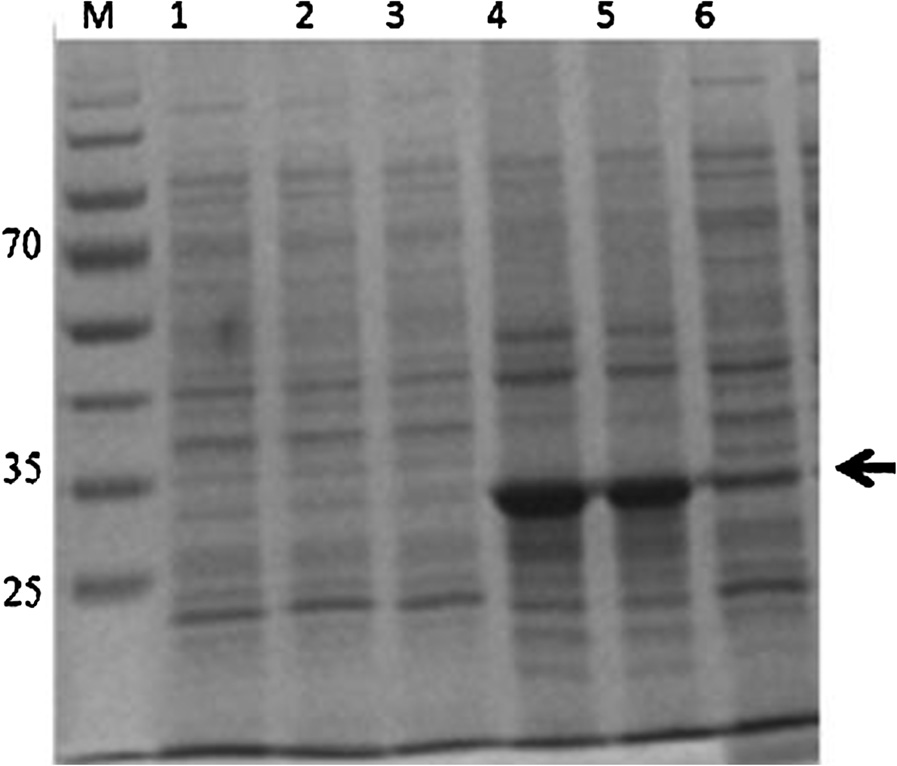
**SDS-PAGE analysis on expression of recombinant *pk*HSPATR protein at fourth hour of induction.** Lane 1-3, Bug Buster extraction of pRSET-B vector protein (control); lane 4-5, Bug Buster extraction of *pk*HSPATR protein; pkHSPATR protein without Bug Buster extraction. Arrow indicates expression of *pk*HSPATR protein at expected size (32 kDa).

Recombinant *pk*HSPATR protein expression was induced using 1 mM IPTG. The protein was expressed four hours after induction and was visible on a 12% Coomassie blue-stained polyacrylamide gel as a 32 kDa band, which was not observed in the negative control (Figure 1). The recombinant *pk*HSPATR was predominantly found within aggregated inclusion bodies. Initially, the cell culture pellet formed a viscous solution when resuspended in PBS and formed insoluble precipitates when boiled with equal volume of 2X loading dye (100 mM Tris-HCL, 4% SDS, 0.2% bromophenol blue, 20% glycerol, 2.5% mercaptoethanol). The recombinant protein was recovered from inclusion bodies by using the Bug Buster protein extraction kit. Also, Benzonase was added to release the target protein and to reduce the viscosity of the extract.

### Purification of recombinant *pk*HSPATR

**Figure 2:**
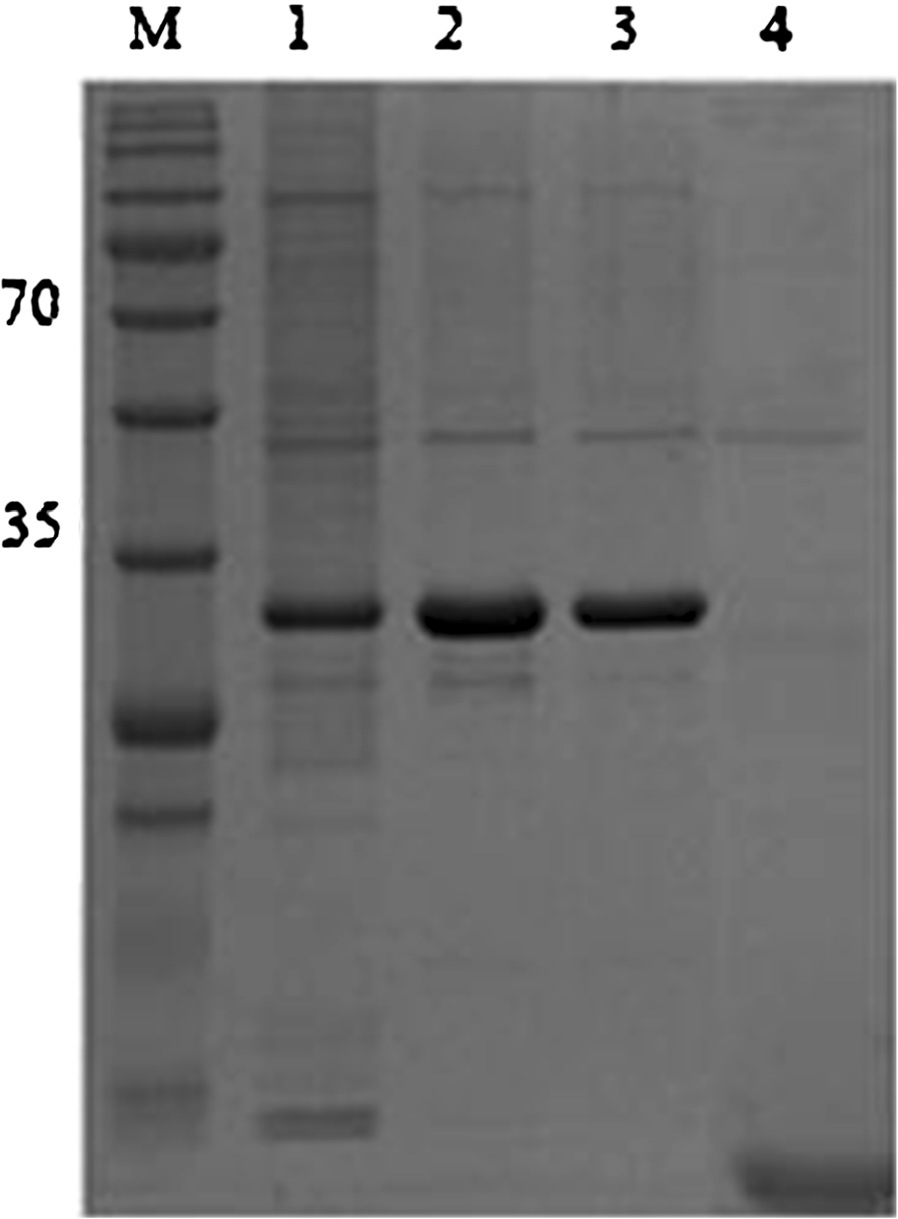
**SDS-PAGE analysis of Ni-NTA purified recombinant *pk*HSPATR protein and vector control.** Lane 1, unbound protein (aspirated supernatant after binding); Lane 2-3, first and second eluate after purification; lane 4, pRSET-B vector protein after purification.

**Figure 3:**
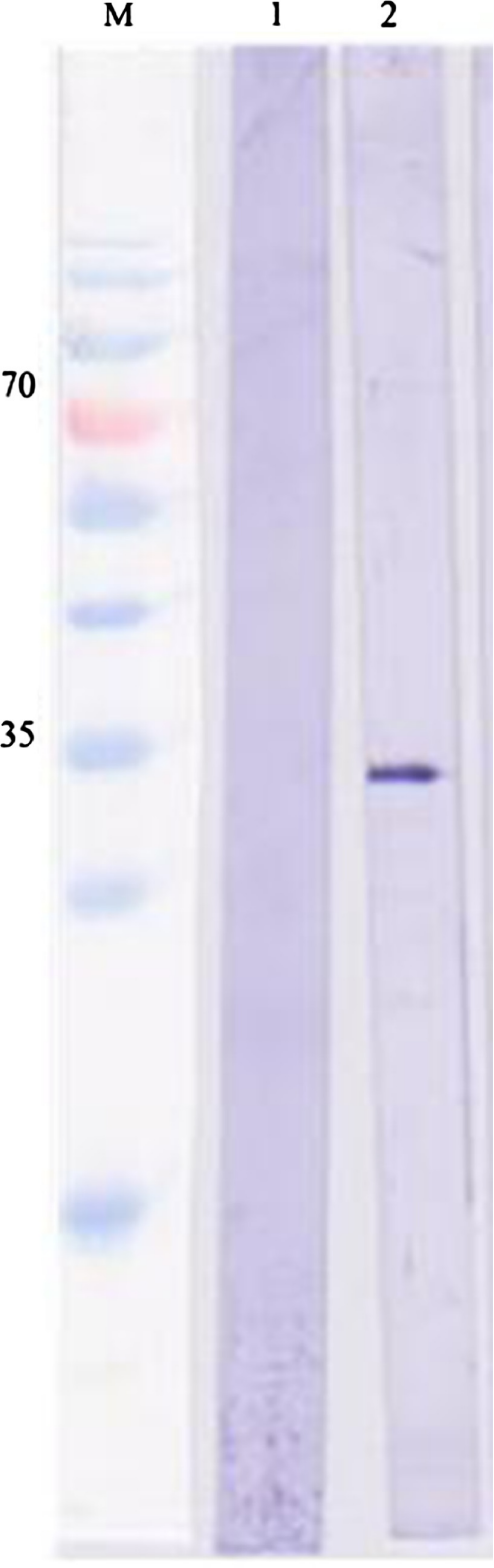
**Immunoblot analysis of Ni-NTA purified recombinant *pk*HSPATR protein and vector protein (control).** Lane 1, pRSET-B vector protein (control) after purification; recombinant *pk*HSPATR protein after purification.

Recombinant *pk*HSPATR was successfully purified under denaturing conditions using Ni-NTA column. The purified protein was run on 12% polyacrylamide gels, and both Coomassie staining and western blot with anti-Xpress revealed a single band at the expected molecular mass of 32 kDa (Figures 2 and 3). Quantification of *pk*HSPATR using Bradford assay yielded a protein concentration of 0.2-0.4 mg/ml.

### Evaluation of sensitivity and specificity of recombinant *pk*HSPATR in detection assays

**Figure 4:**
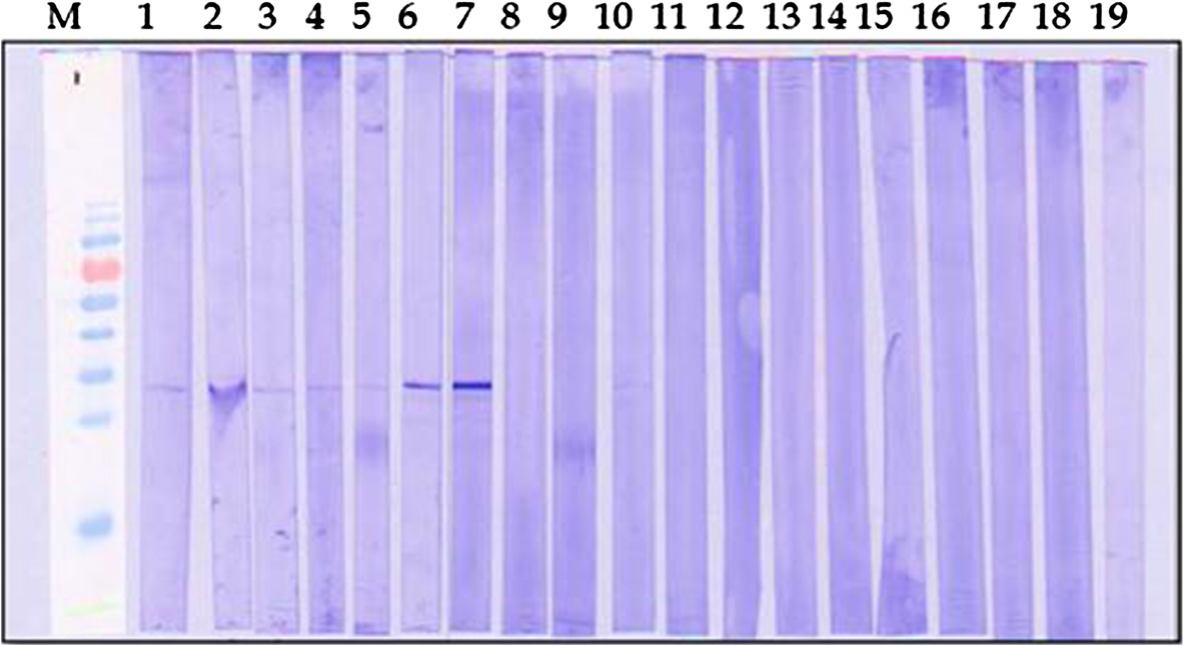
**Detection of recombinant *pk*HSPATR with patient sera infected by *Plasmodium knowlesi *and other parasite species.** Each western blot strip which contained the purified recombinant *pk*HSPATR was tested with serum from each category. Selected samples from each category are shown. Lane M, Bio-Rad Prestained Broad Range Protein Marker; lanes 1 to 7, sera of patients infected with *P. knowlesi*; lane 8-10, serum of patient infected with *P. falciparum*; lane 11-13, serum of patient infected with *P. vivax*; lanes 14-18, sera of patients infected with non-malarial parasites (14- filariasis, 15- amoebiasis, 16- toxoplasmosis, 17- cysticercosis, 18- others), lane 19, healthy donor serum which served as negative control.

The sensitivity and specificity of purified *pk*HSPATR for detection of *P. knowlesi* was evaluated using western blot and ELISA. Human serum samples from four groups were screened: (i) *P. knowlesi*; (ii) non-*knowlesi Plasmodium*; (iii) healthy donors (88 samples); and (iv) non-malaria parasitic infections, including *T. gondii* (14 samples), amoebiasis (three samples), cysticercosis (two samples), and filariasis (two samples). In the western blot assay, 38 out of 40 (95.0%) of the *P. knowlesi-*infected serum samples reacted with the recombinant *pk*HSPATR, yielding a band at ~32 kDa. The two samples not detected by western blot assay were presented with very low parasitemia level, undetectable under microscopy and PCR demonstrated faint bands upon electrophoresis. For non-*knowlesi Plasmodium* samples, five out of 31 (16.1%) of the samples gave a faint positive band. Of these, two samples were from *P. falciparum* infections, while the rest were from *P. vivax* infections. None of the non-malaria and healthy donor serum samples detected a band in the western blot assay (Figure 4). As for ELISA, 35 out of 40 (87.5%) of the *P. knowlesi* samples reacted with *pk*HSPATR, while six out of 31 (19.4%) of the non-*knowlesi Plasmodium* samples reacted with the recombinant protein. No samples from the other infections reacted with the recombinant protein. Finally, the sensitivity (number of true positives/number of true positives + number of false negatives) and the specificity (number of true negatives/number of true negatives + number of false positives) of the *pk*HSPATR-based assays were determined. The calculated values for sensitivity and specificity of *pk*HSPATR in the western blot assay were 95 and 96.7%, respectively. Sensitivity and specificity of *pk*HSPATR in the ELISA-based assay were found to be 87.5 and 96.0%, respectively.

## Discussion

The complex malaria parasite life cycle, which is accompanied by alterations in gene expression at each of its stages, results in major antigenic changes during malaria infection [8]. Logically, the most reliable and effective antigens for diagnosis of *P. knowlesi* infection would be those that are expressed at multiple stages of the parasite developement and preferably associated with invasion, sequestration, and progression of pathogenesis in the host [6]. In this regard, the multistage expressed *spatr* gene of *Plasmodium* contains an altered TSR domain, which has been reported to play a role in parasite mobility, host cell attachment, and host cell invasion [5,6].

Here, cloning and expression of the *spatr* gene from *P. knowlesi* were reported. Moreover, the recombinant *pk*HSPATR protein was purified and tested if it can be utilized to sensitively and specifically detect *P. knowlesi* infection in serum from malaria-infected individuals. In addition, the native *spatr* gene fragment was codon optimized to increase its expression level in *E. coli.* Generally, *Plasmodium* species have higher adenine and thymine (A+T) content in their nucleotide sequences [9,10]. Thus, codon bias of the *Plasmodium* gene within the heterologous expression system could interfere with successful recombinant protein expression due to depletion of rare transfer RNAs. Moreover, global reduction of A+T content can cause mRNA instability, or decrease the rate at which the mRNA is exported into the cytoplasm [10-12]. Nevertheless, using the codon optimized version of the *P. knowlesi pkhspatr* gene, efficient expression was observed within *E. coli* system, allowing successful purification of the *pk*HSPATR recombinant protein.

The *pk*HSPATR recombinant protein was recovered as inclusion bodies from the *E. coli* expression system. The mechanism of inclusion body formation is still not very well understood; however, polypeptide misfolding caused by incorrect disulphide linkage among the 12 cysteine residues within the SPATR protein has been proposed as one of the causes of inclusion body formation [6]. In addition, temperature and pH of the cultivation medium have been suggested as possible contributing factors. Also, it has been hypothesized that inclusion body formation might even stabilize the protein by protecting it from protease degradation. These protein aggregates were once considered to be waste products, but recent discoveries have revealed that inclusion bodies actually contain biologically active polypeptides [13]. The inclusion bodies purified in this study were denatured using the Bug Buster protein extraction kit, and sequential reduction in urea concentration was used to refold the denatured protein [14].

In a previous study, pooled sera from *P. knowlesi-*infected rhesus monkeys were shown to react with recombinant *pk*SPATR protein in ELISA [6]. This finding was extended by systematically testing the sensitivity and specificity of the use of *pk*HSPATR in two detection assays. In fact, the sensitivity of recombinant *pk*HSPATR was differed between western blot and ELISA for detecting antibodies against *P. knowlesi.* In the ELISA assay, the cut-off value was based on an average obtained from sera of healthy donors. However, it is possible that the empirically determined threshold value may have been too high, causing *P. knowlesi*-infected samples that had tested positive by western blot to be considered negative in the ELISA. Moreover, the purity of the recombinant protein might influence the readings obtained in the ELISA, generating false positive results [15,16] for non-*knowlesi* samples. Additionally, ELISA could also underestimate the amount of bound recombinant protein, as only the correctly folded protein might be correctly detected, resulting in reduced sensitivity (Table 1). However, in the western blot assay, boiling and the electrophoresis process denatures the protein, which allows a more sensitive binding of *P. knowlesi* antibodies to linear epitopes.

**Table 1:** **Sensitivity and specificity analysis of recombinant *pk*HSPATR protein against human serum samples using western blot assay and ELISA**

	**Number**				
**Human sera group**	**of sera**	**Western blot**		**Elisa**	
	**Tested**				
		**Positive**	**Negative**	**Positive**	**Negative**
		**No.**	**No.**	**No.**	**No.**
A.*Plasmodium knowlesi*	40	38	2	35	5
B.Non-*knowlesi*					
*Plasmodium*					
i.*P.vivax*	16	3	13	3	13
ii.*P.falciparum*	15	2	13	3	12
iii.*P.ovale*	1	0	1	0	1
C.Non-malaria parasitic					
infection					
i.Filariasis	2	0	2	0	2
ii.Amoebiasis	3	0	3	0	3
iii.Cysticercrosis	2	0	2	0	2
iv.Toxoplasmosis	14	0	14	0	14
v.Others*	11	0	11	0	11
Healthy donors	88	0	88	0	88

*Non-malaria patient presented with fever.

Cross-reactivity of the recombinant protein with serum from individuals infected with other *Plasmodium* species could result from shared sequence homology. Antibodies directed against a specific protein often interact with other closely related proteins with equal specificity. In this regard, there is a conservation in the number and position of all cysteine residues within the SPATR proteins from *P. knowlesi*, *P. falciparum*, *P. vivax* and *P. yoelii *[6]. Also, this similarity might be consistent with previous studies of cysteine-rich malarial proteins, which suggested that they were important for parasite attachment and invasion of host cells. Indeed, SPATR protein has been reported to attach to human liver cells and play a role in hepatocyte invasion by sporozoites.

## Conclusions

In summary, the *pk*HSPATR recombinant protein seems to allow sensitive detection of human *P. knowlesi* infection in serum samples by immunoblot and ELISA. With further research, the *pk*HSPATR protein could be exploited as a sensitive marker for detection of *P. knowlesi* infection in humans; however, this protein remains to be fully immunocharacterized. In addition, findings from this study provide rationale for the production of a specific recombinant antigen for use in immunodiagnostic assays and *P. knowlesi* vaccine development.

## Competing interests

The authors declare that they have no competing interests.

## Authors’ contributions

VP carried out laboratory works. RM conducted clinical diagnosis on patient blood samples. VP, YLL, FMY and CY participated in data analysis. VP and YLL drafted the manuscript. All authors read and approved the final version of the manuscript.
